# Supplementary figures and images for: Toxoplasma gondii PPM3C, a secreted protein phosphatase, affects parasitophorous vacuole effector export
Source: PLoS Pathog. 2020 Dec 28;16(12):e1008771. doi: 10.1371/journal.ppat.1008771 (PMC7793252; doi:10.1371/journal.ppat.1008771)

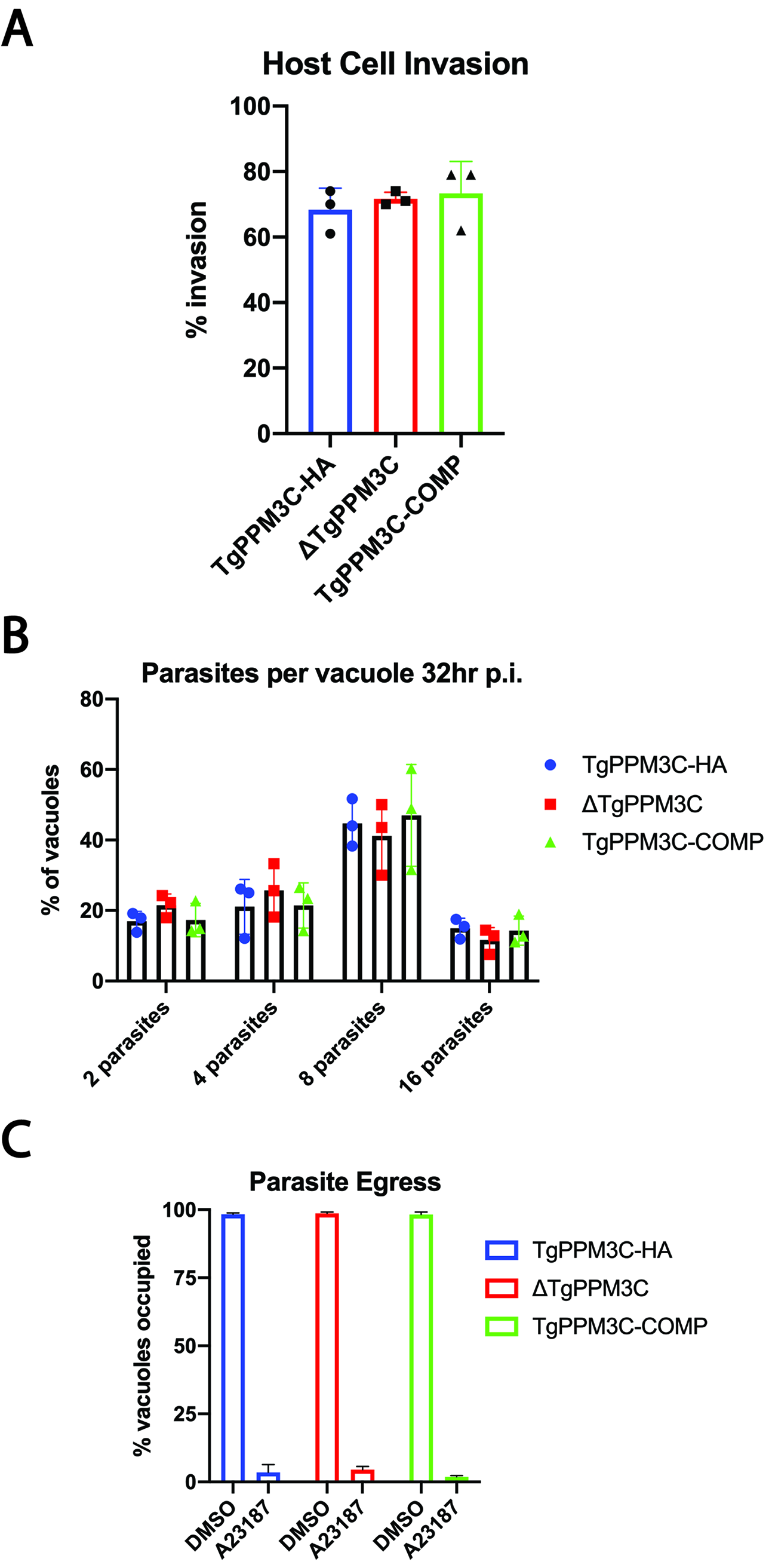

Supplement: S1 Fig — (A) Parasite invasion was assessed by immunofluorescence 30 minutes post-addition of extracellular tachyzoites to an HFF monolayer, using antibodies to label parasites before and after permeabilization of host cells. Data from three independent experiments are shown. No significant differences were measured between the TgPPM3C-HA, ΔTgPPM3C, and TgPPM3C-COMP strains. (B) Parasite replication was assessed by immunofluorescence after 32 hours of infection, counting the number of parasites per vacuole from at least 150 vacuoles of each strain per experiment. Data from three independent experiments are shown. No significant differences were seen between the TgPPM3C-HA, ΔTgPPM3C, and TgPPM3C-COMP strains. (C) Parasite egress was assessed by immunoflourescence after adding either DMSO (control) or the calcium ionophore A23187 and quantifying the number of occupied (GRA6 positive, SAG1 positive) and unoccupied vacuoles (GRA6 positive, SAG1 negative) from at least 200 vacuoles per strain for each condition and experiment. Data from three independent experiments are shown. No significant differences were seen between the TgPPM3C-HA, ΔTgPPM3C, and TgPPM3C-COMP strains. (TIF) [file ppat.1008771.s001.tif]

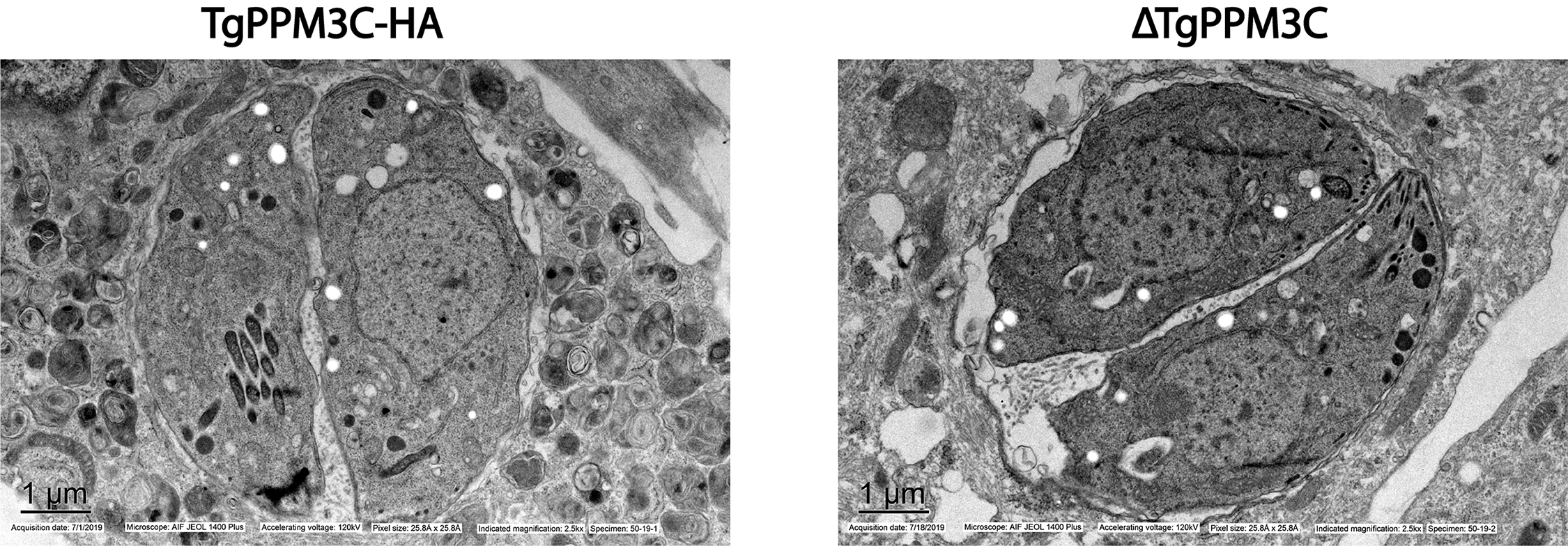

Supplement: S2 Fig — Representative transmission electron micrographs of vacuoles formed by either TgPPM3C-HA or ΔTgPPM3C parasites, 32 hours post-infection in human fibroblast monolayers. Tubules from the intravacuolar network, a hallmark of tachyzoite Toxoplasma vacuoles, can be seen in the vacuoles formed by both strains. (TIF) [file ppat.1008771.s002.tif]

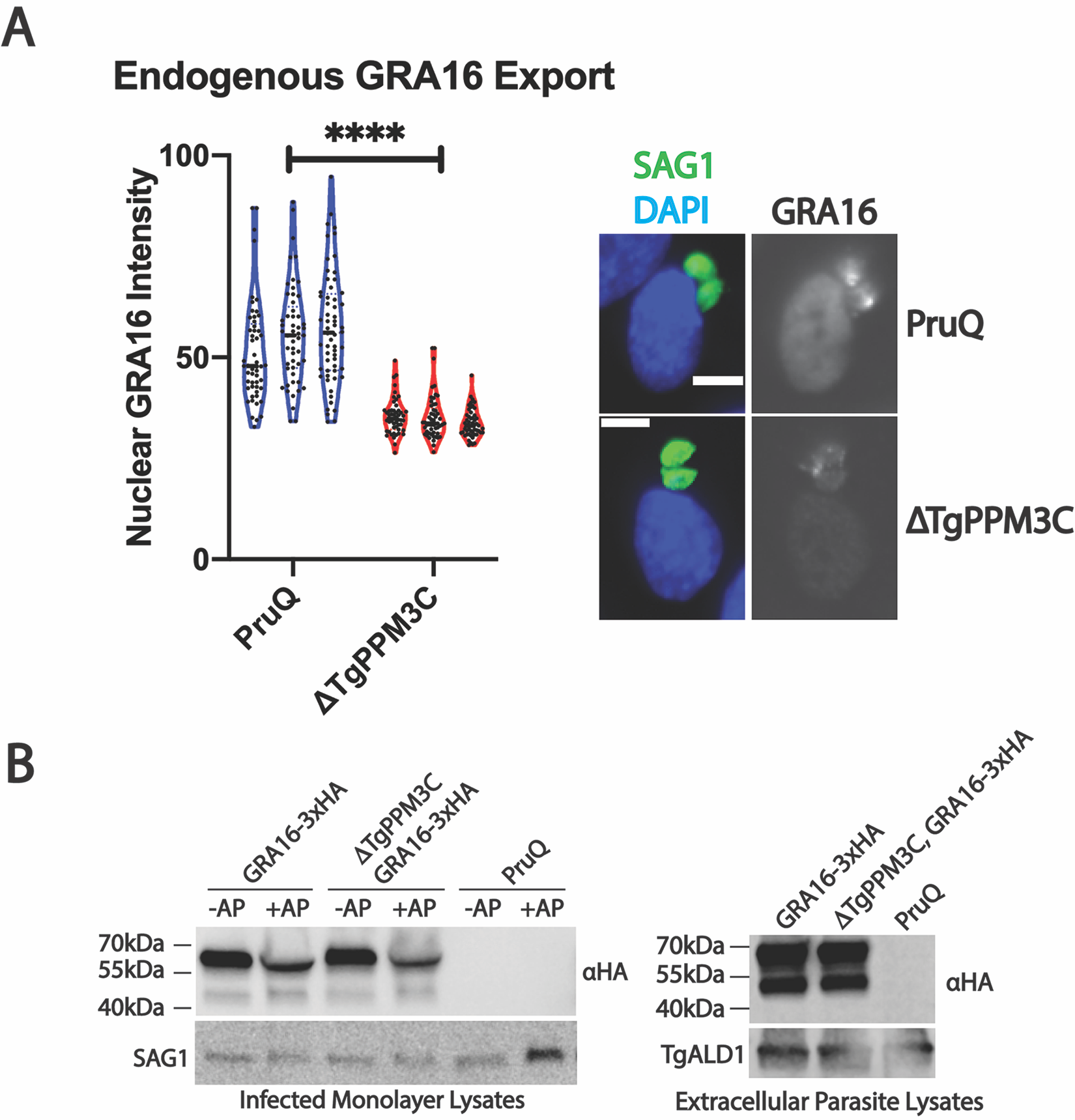

Supplement: S3 Fig — (A) Violin plots of endogenous GRA16-3xHA accumulation in host nuclei infected with either PruQ or ΔTgPPM3C parasites. GRA16 fluorescence was quantified from fibroblast nuclei containing a single parasite vacuole. A significant decrease in GRA16 host nuclear accumulation is observed during ΔTgPPM3C infection, suggesting defects in effector export from the parasitophorous vacuole. Data were collected from three independent experiments. Representative images from which effector intensity were quantified are shown on the right. Antibody to SAG1 was used as a parasite marker, DAPI as a host nucleus marker, and anti-HA antibody was used to detect GRA16-3xHA. Scale bar equals 10μm. (B) Immunoblots of protein lysates obtained from infected monolayers (left panel) or extracellular parasites (right panel). Infected monolayer lysates were treated (+AP) or mock treated (-AP) with 100U of alkaline phosphatase for 1hr in a 37C water bath. A shift in the larger of the two GRA16-3xHA bands (>55kDa) is observed following AP treatment of lysates obtained from both the GRA16-3xHA and ΔTgPPM3C strains, suggesting that this band represents phospho-GRA16. The larger GRA16-3xHA band is also detected in extracellular parasite protein lysates, indicating GRA16 is likely phosphorylated within parasites prior to secretion. Antibody to SAG1 or TgALD1 was used as a loading control. The infected monolayer lysate immunoblot with alkaline phosphatase treatment is representative of two independent experiments. (TIF) [file ppat.1008771.s003.tif]

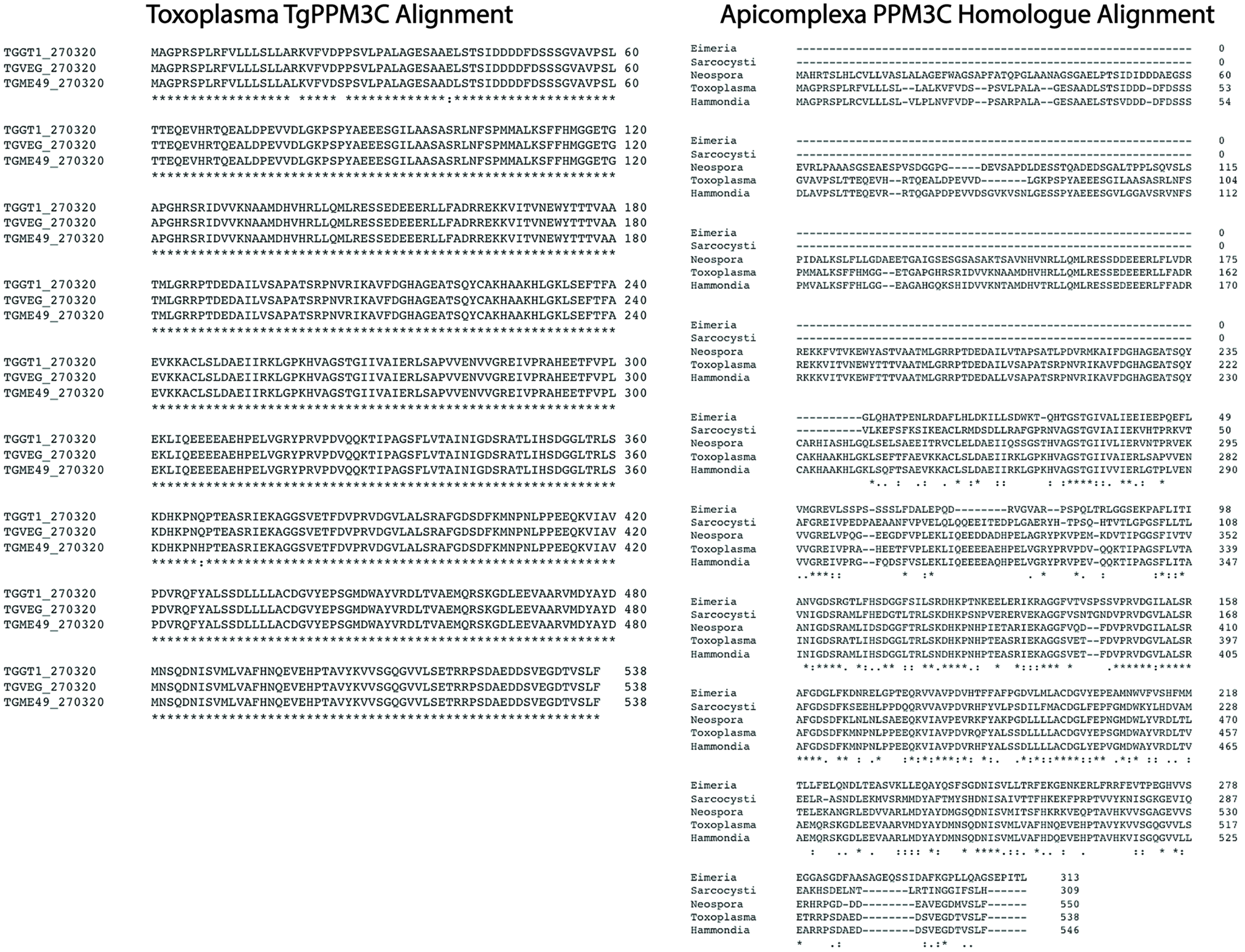

Supplement: S4 Fig — Clustal Omega alignment of TgPPM3C amino acid sequences from the Type I reference strain GT1, Type II reference strain ME49, and Type III strain VEG. An amino acid alignment comparing select Apicomplexan PPM3C homologues is also shown for Toxoplasma strain ME49 (TGME49_270320), Hammondia hommondi strain H.H.34 (HHA_270320), Neospora caninum Liverpool (NCLIV_036340), Sarcocystis neurona SN3 (SN3_02500075), and Eimeria acervuline Houghton (EAH_00048430). Sequences were obtained from ToxoDB and EuPathDB. (TIF) [file ppat.1008771.s004.tif]

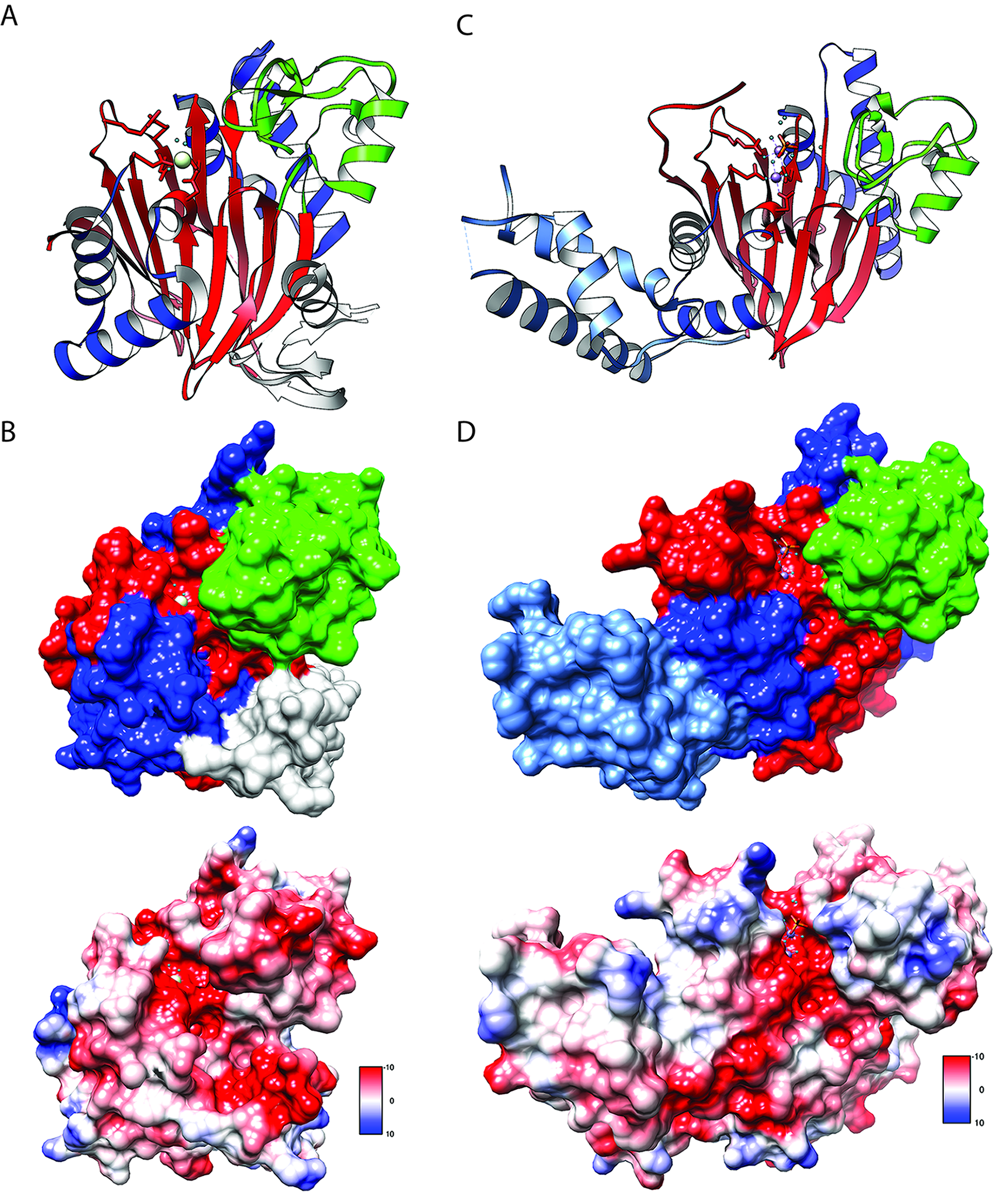

Supplement: S5 Fig — (A) Ribbon projection of TgPPM3C crystal structure. The characteristic beta sandwich (red) formed by adjacent beta sheets can be seen coordinating a Praseodymium cation (Pr3+) (white circle) in the putative active site of the enzyme. The side chains of the conserved metal and phosphate coordinating amino acids are shown, along with water molecules (small cyan circles) in this region. Alpha helices (blue) surround the beta sandwich. The flap domain (green) is seen above the beta-sandwich pocket, along with an additional domain (white) present beneath the flap domain, which is not seen in the human PPM1A crystal structure. (B) Top–surface mesh rendering of TgPPM3C, demonstrating the putative catalytic pocket in which the Pr3+ ion is found. Coloring scheme is as described in A. Bottom–calculated Coulombic potential for the TgPPM3C surface mesh, demonstrating a strongly acidic (red) putative catalytic pocket with no basic residues (blue) near the beta-sandwich cleft. (C) Ribbon projection of the human PPM1A crystal structure. The beta sandwich (red) can be seen coordinating two Mg2+ cations (purple circles) in the active site, along with a phosphate ion (orange stick figure) just above the Mg2+ cations. The side chains of the conserved metal and phosphate coordinating amino acids are shown, along with water molecules (small cyan circles) in this region. The flap domain is shown in green, and the C-terminal domain extending away from the center of the enzyme is shown in light blue. (D) Top–surface mesh rendering of PPM1A, demonstrating a similar active site pocket as seen in TgPPM3C. Coloring scheme is as described in C. Bottom–calculated Coulombic potential for the PPM1A surface mesh, demonstrating the characteristic acidic (red) catalytic pocket and a few basic residues (blue) above the catalytic pocket. Molecular graphics and analyses of crystal structures were prepared using UCSF Chimera [59] and the publicly available structures of the TgPPM3C catalytic domain (Prot [file ppat.1008771.s005.tif]
