# Supplementary material for: Toxoplasma gondii PPM3C, a secreted protein phosphatase, affects parasitophorous vacuole effector export
Source: PLoS Pathog. 2020 Dec 28;16(12):e1008771. doi: 10.1371/journal.ppat.1008771 (PMC7793252; doi:10.1371/journal.ppat.1008771)
Supplement: S1 Table — (DOCX) [file ppat.1008771.s006.docx]

| Name | Sequence (5’ – 3’) | Purpose |
| --- | --- | --- |
| gRNA_R | AACTTGACATCCCCATTTAC | KLD sgRNA Cloning |
| KLD_PPM3C_gRNA_FWD | GCGGAGGACGACAGCGTCGAGTTTTAGAGCTAGAAATAGC | KLD sgRNA Cloning |
| KLD_PPM3C_KO_FWD | GGGACCCGCCATCTTTCCAGGTTTTAGAGCTAGAAATAGC | KLD sgRNA Cloning |
| KLD_COMP_gRNA_FWD | TGACTAACTAGCTAACTAGGGTTTTAGAGCTAGAAATAGC | KLD sgRNA Cloning |
| PPM3C_CTermHA_FWD | GGAAGACGACAGCGTGGAGGGCGACACAGTTTCCCTGTTTTACCCTTACGATGTACCGGA | C-terminal tagging |
| PPM3C_CTermHA_RVS | TCTTCACTGTCGTTCACAGTCAACAAAAGTCAAGTCCCATCTCTTCGCTATTACGCCAGC | C-terminal tagging |
| PPM3C_KO_Donor_FWD | TATTCGCCTTCTGATTTGTCTCGACCGCCTCTGGAAAG TGACTAACTAGCTAACTAGGGGGGCGGGTCCCCGATCTCCTCTCCGCTTTGTTCTTCTTCTC | KO donor oligo |
| PPM3C_KO_Donor_RVS | GAGAAGAAGAACAAAGCGGAGAGGAGATCGGGGACCCGCCCCCCTAGTTAGCTAGTTAGTCACTTTCCAGAGGCGGTCGAGACAAATCAGAAGGCGAATA | KO donor oligo |
| PPM3C_COMP_  Donor_FWD | CCCATCCCCTGTTATTCGCCTTCTGATTTGTCTCGACCGCCTCTGGAAAGATGGCCGGCCCCCGATCTCCTCTCCGCTTTGTTCTTCTTCTCTCTCTACT | COMP donor oligo |
| PPM3C_COMP_  Donor_RVS | AGTAGAGAGAGAAGAAGAACAAAGCGGAGAGGAGATCGGGGGCCGGCCATCTTTCCAGAGGCGGTCGAGACAAATCAGAAGGCGAATAACAGGGGATGGG | COMP donor oligo |
| GRA16_CTerm_3HA_FWD | TTTTGACTGTTCACGCGCGAAGCGGAAAAATGATCAGATGTACCCGTACGACGTCCCGGA | C-terminal tagging |
| GRA16_CTerm_3HA_RVS | TGCCAGCACGTGGGTACCGTCCGAGAGAGGCGTGTAGCATCCTTCTGGTTAGTTAGGCATAATCTGGAACAT | C-terminal tagging |
| pLIC_3HA_1_FWD | AGGTACCCGTACGACGTC | Gibson Assembly |
| pLIC_3HA_2_RVS | TTCCGAGCTTGGCGTAATCA | Gibson Assembly |
| GA_GRA16_  Promoter_FWD | TGATTACGCCAAGCTCGGAATGAGAAACGGTTATCCTGAT | Gibson Assembly |
| GA_GRA16_  CTerm_RVS | GGGACGTCGTACGGGTACCTCATCTGATCATTTTTCCGCT | Gibson Assembly |
| GA_GRA24_  Promoter_FWD | TGATTACGCCAAGCTCGGAATGCTGGCCATTCATCTTTAC | Gibson Assembly |
| GA_GRA24_  CTerm_RVS | GGGACGTCGTACGGGTACCTATTACCCTTAGTGGGTGGTT | Gibson Assembly |
| GA_GRA28_  Promoter_FWD | TGATTACGCCAAGCTCGGAAGTGACGGCGCTTCTCCTGAC | Gibson Assembly |
| GA_GRA28_  CTerm_RVS | GGGACGTCGTACGGGTACCTTTCGGAATAACTGGAGCTAC | Gibson Assembly |
| GA_TgIST_  Promoter_FWD | TGATTACGCCAAGCTCGGAAAAAAGTTCCTCTGGAATCTG | Gibson Assembly |
| GA_TgIST_  CTerm_RVS | GGGACGTCGTACGGGTACCTGACCCGCCGTTCACTACTCG | Gibson Assembly |
| KLD_GRA16_S97E_FWD | TTCTCCTGGCGAGCTGGGCGGTT | GRA16 Mutagenesis |
| KLD_GRA16_S97P_FWD | TTCTCCTGGCCCGCTGGGCGGTT | GRA16 Mutagenesis |
| KLD_GRA16_S97_RVS | GTCGTCCTGGAACCTTGAGC | GRA16 Mutagenesis |
| KLD_GRA16_S148E_FWD | GCCTCAATATGAGCAAACAGATG | GRA16 Mutagenesis |
| KLD_GRA16_S148P_FWD | GCCTCAATATCCGCAAACAGATG | GRA16 Mutagenesis |
| KLD_GRA16_S148_RVS | CTGGTTCTACGAAGCTTCAA | GRA16 Mutagenesis |
| PPM3C_CTerm_FWD | AAACTTTGTGTCTTCTCTGT | Confirm gDNA editing |
| PPM3C_3Flank_RVS | TCCATCCCGATGTACCCGCC | Confirm gDNA editing |
| PPM3C_5UTR_FWD | TGCAAGACCTTCGTCCCTGT | Confirm gDNA editing |
| PPM3C_NTerm_RVS | GACACCCGAGGAACTGTCGA | Confirm gDNA editing |
| GRA16_CDS_FWD | AGTACCAGCTCAGGTGCAGC | Confirm gDNA editing |
| GRA16_3UTR_RVS | CACTGCCTCACATTTGCAGT | Confirm gDNA editing |

**Supplemental Table 1.** Primers used for sgRNA cloning, PCR amplification of C-terminal tagged donor sequences, Gibson Assemblies, GRA16 mutagenesis, and confirmation of genomic DNA (gDNA) editing.

-For primers with overhanging sequences, homology to the plasmid template is designated in blue.

-Mutations preventing Cas9 cleavage of donor sequences are designated in red. Multi-stop codon sequence/handle is also designated in red, as well as mutations for GRA16 mutagenesis. In coding regions, all point mutations for Cas9 editing were designed to be synonymous.
