# Supplementary material for: Toxoplasma gondii PPM3C, a secreted protein phosphatase, affects parasitophorous vacuole effector export
Source: PLoS Pathog. 2020 Dec 28;16(12):e1008771. doi: 10.1371/journal.ppat.1008771 (PMC7793252; doi:10.1371/journal.ppat.1008771)
Supplement: Attachment — Submitted filename: Response Letter final version V2.docx [file ppat.1008771.s009.docx]

**Reply to the Review**

*We first would like to thank the editor and reviewers for their thoughtful comments, and for considering this work as valuable and of interest to the Toxoplasma field. We were particularly grateful for the detailed suggestions on how to improve this manuscript and how to more clearly state reasons for different observations between this study and those in a recent and highly relevant study (Cygan et al.). We have provided a detailed response to the various reviewer's comments below in 12pt italicized font. We believe that the new experimental data and revisions have significantly improved this manuscript and that this work merits publication in PLoS Pathogens. All of the authors agree with submission of this revision and in the event of publication the authors assign copyright in accordance with the instructions to authors.*

**Response to the Editor's comments**:

The reviewers have mixed opinions regarding the paper although I believe that this represents a significant advance for the field. But, the reviewers raise important concerns/points. While all of their comments should be addressed, particular attention should be paid to: providing higher quality immunofluorescence images, defining which lytic growth step is affected by loss of PPM3c, examining, if possible, the impact of PPM3c on Myr1 phosphorylation and interactions, addressing why the phosphomimetic and phosphoablative GRA16 mutants have similar phenotypes, and better integrating this work with that of Cygan et al.

*We appreciate the editor's comment that this is a significant advance for the field. In this revised manuscript, we have addressed the comments of the reviewers as suggested and performed additional experiments to provide higher quality immunofluorescence images, examine which lytic growth step is affected by loss of PPM3c, addressed the impact of PPM3c on Myr1 phosphorylation and interactions, and addressed why the phosphomimetic and phosphoablative GRA16 mutants have similar phenotypes. In addition, we have provided more discussion and integration of our findings and the data presented in Cygan et al.*

**Response to the reviewer’s comments:**

**Part I Summary (**Please use this section to discuss strengths/weaknesses of study, novelty/significance, general execution and scholarship)

Reviewer #1: Mayoral et al investigate the role of the secreted phosphatase PPM3C during Toxoplasma infection. They show this PV/cyst-localised phosphatase has a critical role in acute infection and a modest growth defect in vitro. Using co-IP and phosphoproteomics to identify interaction partners/targets they identify both translocon component and known secreted proteins. PPM3C dependent defects in secretion are subsequently shown for GRA16 and GRA28, although importantly not for IRF1 phenotype that is dependent on TgIST. Phosphomutants of GRA16 further indicate a role of phosphoregulation in the secretion of this protein. Overall, I think this is an interesting piece of work and a valuable contribution to the field. While it was shown in 2011 that many secreted proteins are phosphorylated, further developments in our understanding have been limited and the critical role of PPM3C during infection and involvement in protein secretion into the host cell is noteworthy. However, I was disappointed that the MYR1 phosphorylation was not investigated further as insight into regulation of the translocon could be more insightful than individual effectors. Additionally, PPM3C localisation has been previously reported and its contribution to effector translocation investigated yet the authors did not discuss these conflicting results.

Reviewer #2: Toxoplasma can export GRA effectors beyond the parasitophorous vacuole membrane. These GRA effectors play important roles in the modulation of host cell signaling pathways and the host immune response. Therefore identifying the exact mechanisms of GRA translocation are important. Multiple Toxoplasma proteins have now been identified that affect GRA effector translocation, some of these are part of the translocon while others seem to affect the correct folding or phosphorylation status of either translocon components or of the secreted effectors. Here Mayoral et al. show that the PPM3C secreted protein phosphatase affects the export of some GRA effectors likely by affecting their phosphorylation status. They used multiple independent methods to show that it is likely that PPM3C affects the export of GRA effectors by modulating their phosphorylation status. Overall the experiments were well performed and the conclusions valid. This manuscript will be of interest to the field.

Reviewer #3: The goal of this paper is to follow up on a putative phosphatase (TgPPM3C) identified from a prior proteome study of cyst wall. Here the authors seek to show the effect of TgPPM3C on the phosphostate of Toxoplasma proteins, and how this impacts 2 dense granule proteins that are exported via the MYR1-complex. The strength of the paper is the concept that phosphostate (via TgPPM3C) might affect dense granule protein secretion through the MYR-complex. Unfortunately, the data are not convincing and contradict a recently published paper (ref 16) that used more rigorous methods.

*We appreciate each of the reviewer’s overall insights and impressions. Below we attempt to justify our conclusions both by significantly revising the manuscript text as well as through the inclusion of additional experiments to enhance the rigor of our findings. As will be stated several times below, we do not believe our findings contradict the data in Cygan et. al. Cygan et al., probed for the presence or absence of both host c-Myc induction and GRA16 export, while we quantitatively measured host nuclear c-Myc and host nuclear GRA16 fluorescence intensity. Our findings are in agreement with Cygan et al. in that deletion of TgPPM3C does not abrogate host c-Myc or GRA16 export completely, but they also demonstrate that TgPPM3C appears to influence these processes (as demonstrated in our data using the various genetically modified Pru strains characterized in our manuscript). In addition, it is possible that the use of different strains in each of our respective works (Type I RH strain in Cygan et. al. vs. Type II Pru strain in this study) could also be a factor for any differences. We do not exclude the possibility that subtle differences might also be detected in deltaPPM3C RH parasites if GRA16 export was quantified by the methods described in our manuscript.*

**Part II – Major Issues: Key Experiments Required for Acceptance (**Please use this section to detail the key new experiments or modifications of existing experiments that should be absolutely required to validate study conclusions. Generally, there should be no more than 3 such required experiments or major modifications for a "Major Revision" recommendation. If more than 3 experiments are necessary to validate the study conclusions, then you are encouraged to recommend "Reject".)

Reviewer #1: Novelty: The authors don’t mention that PV localisation and interaction with MYR1 have been previously reported for this protein (despite referencing the paper to say it supports their c-myc experiment). This previous paper uses a different strain of Toxoplasma (RH) but shows that PPM3C KO has no effect on parasite growth, GRA16 nuclear translocation or cMyc upregulation. While there could be strain differences, I think it is essential that the authors discuss rather than ignore these previous results.

*We appreciate this comment on the findings regarding TgPPM3C in Cygan et al., mSphere, 2020. We have provided increased discussion of the interpretation of our findings compared to those in the afore-mentioned study in the revised manuscript. This includes text explicitly acknowledging the findings in Cygan et. al. throughout the results and discussion sections. We believe that the work described by Cygan et al. is important and apologize if reviewer's thought we had not sufficiently acknowledged this work in our previous version of this manuscript.*

Fig 2. In these initial experiments the strains TgPPM3C-HA, deltaTgPPM3C, and TgPPM3C-COMP are used whereas later Pruku80 is used with deltaTgPPM3C. Is there any difference between TgPPM3C-HA and Pruku80 in the plaque assay or GRA16 translocation/c-myc experiments?

*We have not found any significant differences in growth between the TgPPM3C-HA and the Pruku80 (PruQ) strain both during in vitro culture (as measured by plaque assay) as well as when comparing LD50 in C57Bl/6 mice (following intraperitoneal injection), suggesting that the inclusion of an HA tag at C-terminus of TgPPM3C does not adversely affect parasite growth. For this reason, we used the TgPPM3C-HA and Pruku80 strain interchangeably in later experiments, particularly in IFA experiments involving the detection of HA-tagged effectors (which would be confounding if performed in the parental PPM3C strain, as this strain expresses a PPM3C-HA protein).*

Did the authors look in to the MYR1 phosphorylation sites ie whether phosphomutants impact effector translocation? Given this is the only protein identified in both the co-IP and phosphoproteome, this seems central to the story to follow up. Alternatively, as an interaction between MYR1 and GRA16 has previously been shown, have the authors investigated whether GRA16 phosphomutants impacts this?

*These are both interesting questions. While we agree that interrogating the functional consequence of phosphorylation on MYR1 is of interest, at this time we are unsure which phosphomodification would be worthwhile to mimic by site-directed mutagenesis, since to our knowledge no particular region in MYR1 has been shown to be critical in facilitating effector export (in contrast to the R1/R2 regions in GRA16 described in the manuscript). To that end, mutating all four MYR1 phosphosites detected as significantly more abundant in the deltaPPM3C samples would be a significant investment of time and effort. Furthermore, mutating MYR1 residues individually might not yield significant differences in effector export. We agree that this is a logical follow-up to the results we are reporting and is worth pursuing as a future area of investigation in this evolving story.*

*We appreciate the comment on protein interactions of GRA16 and MYR1, as we did not consider determining if protein-protein interactions between the modified GRA16 protein and MYR1 could explain the result in Figure 5B. We examined our pull downs of the epitope tagged GRA16 protein (both mutagenized and mutagenized) or epitope-tagged MYR1 protein and probing for both proteins in the eluates after Co-IP. While we did not identify clear differences in GRA16-MYR1 interaction when comparing wild type and mutagenized GRA16, the GRA16-MYR1 interaction does appear to be perturbed when comparing wild type and deltaPPM3C parasites based on results from MYR1-Myc pulldowns (Fig S3C). Thus, while altered MYR1-GRA16 interactions likely contribute to the GRA16 export defect observed in deltaPPM3C parasites (Fig S3A), we speculate that mutagenized GRA16 protein may also have a perturbed interaction with other components of the putative translocon aside from MYR1. We have added these issues into the discussion in the revised manuscript.*

Reviewer #2: 1) The PPM3C parasites seem to grow slower based on the plaque assays. For protein export were vacuoles with similar number of parasites/vacuole elected for nuclear quantification of exported proteins? It seems that the reduced growth could affect this quantification. It appears that the authors were careful to select only host cells with a single vacuole. Maybe they can go back to the images and at least investigate if the parasites/vacuole influenced these values.

*As requested, we performed a separate experiment to inspect parasites per vacuole among the different strains at 32hrs p.i. The results showed no significant replication defect in the deltaPPM3C strain, indicating that reduced parasite replication is not responsible for the changes in protein effector export observed. Furthermore, no defects in the export of other effectors, such as GRA24 or TgIST, were noted after 24 hrs of infection, which might be expected if a growth defect were responsible for effector export i.e. the export of all effector proteins would be decreased globally if solely due to parasite growth. We make note of this interpretation in the discussion section of the revised manuscript*

2) It was a bit unclear how the statistical analyses on the quantifications of the IFAs (and plaque areas) performed by the authors were performed. The authors state that the number of observations made for quantification are provided above each violin plot and Kruskal-Wallis tests were performed to calculate p-values. From the material and methods it was unclear if they did the statistical analysis on the three means from the three independent biological experiment or if they used all the data from all the individual cells. Individual host cells are not independent observations in a single biological experiment. It seems more correct to use the average of each biological experiment as one observation and perform the statistics on the 3 averages from the 3 independent biological experiments (but maybe this is what they did?). Can the 3 independent averages be indicated in the violin plots?

*In the original version of this manuscript, three replicates were pooled together to present the graphs in a more concise manner, although statistics were performed using the averages from three independent biological experiments. For clarity, data from the three independent experiments are now provided as violin plots for each figure with IFA data and for the plaque assay data.*

3) It was unclear why the phosphomimetic and phosphoablative mutations in GRA16 both showed reduced nuclear export. Maybe the authors could perform these experiment in the knockout and complemented PPM3C strain and show that in the knockout there is no difference in the amount localized to the host nucleus.

*We thank the reviewer for suggesting this clever experiment. The experiment was performed as suggested, transfecting the deltaPPM3C strain with each of the three constructs and measuring nuclear GRA16 intensity in three independent experiments. We felt including the PPM3C-COMP strain was unnecessarily laborious, since the goal of this experiment was to determine whether any differences would be seen in the export of the mutagenized GRA16 proteins in the PPM3C knockout strain compared to the wild-type GRA16 (the hypothesis being that no differences should be seen if wild-type GRA16 in the deltaPPM3C strain is not dephosphorylated, or at least differences in export between the three constructs should be observed to a lesser extent in deltaPPM3C parasites to that seen in wild-type parasites). Importantly, the results show that no significant differences are evident in the export of wild-type and mutagenized GRA16 in the deltaPPM3C background (Fig 5C), indicating that the findings observed in the Pruku80 background are likely not due to an inherent instability of the mutagenized constructs within the host cell nucleus or the parasitophorous vacuole lumen.*

Reviewer #3: 1. Fig 1B, C, D- the staining is very poor quality and the single images are concerning for the HA tag having screwed up the protein’s localization. In particular, C,D do not look like any typical pattern of staining I have seen for proteins secreted into the vacuole. The authors will need to show more convincing data that this protein is in dense granules/secreted into the PV (could the it be ending up in the residual body?) (see ref 16 Fig2B for more typical staining and Fig S2 for staining in the parasite)

*The IFAs were repeated and images were collected with a confocal microscope to better resolve signals and this new images are provided as requested. Vacuolar localization under bradyzoite growth conditions was apparent, whereas under tachyzoite growth conditions, most of the signal appears to be detected within the parasites themselves, with some occasional punctate signals seemingly within the vacuolar compartment. We believe tachyzoite vacuolar PPM3C-HA localization is not as obvious compared to that demonstrated in Cygan et al., 2020 due to the use of a single HA epitope tag at the endogenous locus (unlike the images shown in Cygan et al., 2020 where PPM3C was endogenously tagged with 3 copies of the HA tag). This interpretation is provided in the main text.*

2. Fig 2B- since there a reasonably impressive in vitro defect, the authors should determine if the defect in growth is at the level of attachment/invasion, replication, or egress, especially as in Fig 3, the authors point out that knocking out TgPPM3C affects the phosphorylation state of proteins in organelles involved in most of those processes. (FYI- phenotype different than what was seen in ref 16)

*We performed the suggested experiments but found no significant differences in the deltaPPM3C strain with respect to host cell invasion, parasite replication, or egress efficiency (Fig S1). This data is now provided in the revised manuscript. We describe in the main text that the reasons for the plaque assay phenotype may be due to more subtle reasons not elucidated by the three different assays performed to identify the defect (i.e. inefficient control of host cell signaling or other vacuolar abnormalities that did not overtly affect the number of parasites/vacuole at the timepoints inspected).*

3. Fig 4A,B- This paper finds a mild phenotype with PPM3C KO but ref 16 found no phenotype on Gra16 translocation. These experiments would need to be repeated with a stable GRA16-HA line (akin to ref 16) to confirm these differences because the MYR-machinery/phenotypes are predicted to be conserved between RH (ref 16) and Pru (current paper)

*As requested, we repeated the experiment with parasites expressing endogenously tagged GRA16-3xHA protein both in the Pruku80 and in the deltaPPM3C Pruku80 background (supplementary figure S3). The results also demonstrate an export defect in deltaPPM3C parasites (seemingly even more pronounced than in Fig 4), providing further support for the findings obtained from transiently transfected parasites described in Fig 4.*

4. Fig 4C- to show that TgIST is not affected by the loss of TgPPM3C, they need to do the same type of experiments as in Fig A (transient expression of an epitope-tagged TgIST)

*We performed the suggested experiment not only for TgIST, but also for GRA24. The results indeed confirm the lack of an apparent export defect for the effectors GRA24 and TgIST in the deltaPPM3C strain compared to the wild-type strain. Furthermore, we probed for TEEGR/HCE1 export indirectly by assessing host cell upregulation of EZH2, which is known to be dependent on the vacuolar export of this effector. The results showed no significant difference in EZH2 induction during infection with the deltaPPM3C strain.*

5. Fig 5- Re-do the experiments with a glutamine substitution which most closely mirror the phosphomimetic glutamate substitution. Figure out a way to show that these substitutions don’t just “break” GRA16. The current data could be interpreted as both glutamate and proline disrupt GRA16’s export (or the protein itself) in a way that has nothing to do with the phosphostate.

*We disagree with the notion that a glutamine substitution would “most closely mirror the phosphomimetic glutamate substitution”. The amino acid substitution we used in our studies is commonly used, and widely accepted, for this type of phosphomimetic work. An amino acid with a negatively charged R-group i.e. aspartic acid would most closely mirror the properties of glutamate. Regardless, we feel that exploring the effects of various amino acid substitutions on S97 and S148 GRA16 residues is outside the scope of this work and would be better addressed in future studies, perhaps complementary to structural studies on the “MYR” translocon". Construction and characterization of glutamine substitutions represents a significant amount of additional work that is highly unlikely to alter what is presented in this manuscript.*

*We show in Figure 5 that the greatest defect in apparent GRA16 export was observed when introducing phosphomimetic substitutions (a defect that was significantly greater than that observed for the proline substitutions), which we believe provides valid support for a model in which phosphorylated GRA16 is not exported from the vacuole as efficiently.*

*We did discuss the possibility that both mutagenized forms of GRA16 may somehow be inherently less stable (or “breakable”) than wild-type GRA16 in lines 255-259 of the original manuscript. We have provided new data and discussion in the revised text that there are no significant differences in export between wild-type or mutagenized GRA16 in the deltaPPM3C strain (Fig 5C), providing independent support against the notion that the mutagenized GRA16 proteins studied here are less stable than unmodified GRA16 in either the host cell nucleus or parasitophorous vacuole lumen.*

**Part III – Minor Issues: Editorial and Data Presentation Modifications** (Please use this section for editorial suggestions as well as relatively minor modifications of existing data that would enhance clarity)

Reviewer #1: Fig1. Please add comment that PV localisation has been shown. Additionally, do you have any hypotheses as to why this looks different? Do the authors ever see signal throughout the vacuole rather than the aggregation you show? Whilst MYR1 is not always PVM localised, how would they envisage this aggregation of PPM3C you show fits with dephosphorylating MYR1 and secreted proteins?

*See above response to 1^st^ major issue raised by Reviewer #3. We collected images that were more representative of TgPPM3C-HA localization and acknowledge the previously described vacuolar localization of TgPPM3C-3xHA from Cygan et al. in the revised text.*

From the Fig. 2D images the larger plaques present in TgPPM3C-HA but not deltaTgPPM3C look like merged neighbouring plaques. How do they account for this in the analysis? Do they have corresponding data on the number of plaques per strain (looks reduced)? As before please add comment on the difference to RH data. Do the authors think this is a true strain difference or due to experimental differences?

*Images from plaque assays were all analyzed blindly and we separated plaques that appeared to arise from two or more merged plaques with the line tool in ImageJ prior to quantifying plaque size for every image/well. We noted no significant differences when assessing the number of plaques between strains across independent experiments. We included more information on plaque assay analysis in the Materials and Methods section for clarity and also added comments on the different result obtain in RH in Cygan et al. in the Discussion section. In the dicussion we interpret these observations as possible evidence for a more heavy reliance on TgPPM3C activity in the Pru Type II strain compared to the more virulent Type I RH strain.*

Table 1. The co-IP experiment gave very few interacting partners. Whilst the short-term interactions of the enzyme could explain this I was surprised by the use of sonication in the protocol. Is this routine in their lab? I thought this high energy treatment disrupted many protein:protein interactions (even if an ice bath is used)? Also, please add comment that MYR1-PPM3C interaction was previously reported.

*We adapted the sonication step from the Co-IP protocol described in Marino et al., PloS Pathog, 2018, where an interaction was preserved at least between MYR1 and MYR3. We felt the sonication step was necessary to fully solubilize material prior to incubation with antibody coated magnetic beads, which in our experience tends to also non-specifically collect bulk material (hence the inclusion of a low-speed centrifugation step to clear the lysates prior to overnight antibody-coated bead incubation). We included comments and references in the Results and Discussion sections on the previously reported interaction between MYR1 and TgPPM3C in Cygan et al.*

It would be good to see TgIST nuclear localisation data in the PPM3C KO (currently data not shown) with only the IRF1 phenotype shown in the Figure. Could there be slightly reduced TgIST secretion and still observe the IRF1 as analysed here? (Ie perhaps surplus is secreted)

*As requested we have now included data on TgIST and GRA24 host nuclear intensity in PruQ and deltaPPM3C strains from three independent experiments (Fig 4A). The data demonstrate no significant differences between the two strains, demonstrating a concordance with the observations made on IRF1 suppression.*

Fig 4 GRA16 and GRA28 nuclear translocation: The PPM3C KO looks like single parasite vacuoles with the SAG1 staining in contrast to a 4 pack with the PruQ. Was there a difference in growth rate with the PruQ – singles/double parasites seem very low for a 24h timepoint. Have you tried normalising the nuclear signal to that in the vacuole to help account for these differences? Otherwise could the reduced nuclear level not reflect the reduced protein in the vac? And again, the contrasting phenotype to the published RH PPM3C KO needs discussed.

*As demonstrated in Fig S1, we found no differences in parasites/vacuole when comparing the various strains at 32 hours post-infection (Fig S1). Similarly, no significant differences were observed when comparing PruQ vs. deltaPPM3C parasites/vacuole in the images used for nuclear effector quantitation (a different representative image was selected to reflect this for GRA16 export in Fig 4A). deltaPPM3C parasites with intense GRA16 and GRA28 vacuolar staining were also observed in the data obtained for Fig 4A. Hence, given these findings, we interpret the data as reflecting a selective export deficiency as opposed to a reduced amount of vacuolar GRA16 and GRA28 compared to the other effectors.*

*In summary, as described in the revised manuscript, we do not believe our results are in disagreement with the observations made in Cygan et al. regarding c-Myc induction and GRA16 export, as we also did not find TgPPM3C to be essential for these processes. Whereas Cygan et al., quantified only the presence or absence of these phenotypes during infection, we quantified the extent of GRA16 export and host c-Myc induction by fluorescence intensity in host nuclei.*

*We do not exclude the possibility that subtle differences might also be detected in deltaPPM3C RH parasites if quantified in this manner.*

**Minor comments:**

I found that both the introduction and discussion were overly focussed on chronic infection and lacked other key information while the data addressed acute infection – for example writing more regarding cyst wall components rather than commenting secreted proteins are extensively phosphorylated (after secretion). Whilst I understand the cyst wall proteome led them to this phosphatase, this could be explained in a sentence. Additionally, chronic kinases are mentioned with more emphasis than the PV localised WNG1 that impacts membrane structures in the PV and is implicated in the phosphorylation of some of the targets identified.

*The reviewer is correct that the majority of the data we have presented were obtained by studies on tachyzoites rather than bradyzoites. As requested we have modified both the introduction and discussion to decreased the number of sentences and to place less emphasis on the phosphorylation of proteins within tissue cysts during chronic infection devoting the majority of the discussion text on vacuolar protein phosphorylation in the context of tachyzoite vacuoles..*

Lines 128-132, Fig 2C/D and legend
The figure legend and methods are not consistent on the inoculum for the cyst counting experiment. Please check – and having the number in the main text would be more helpful than ‘equal inoculums’, especially as the information is there for the survival experiment.

*We thank the reviewer for catching this discrepancy and changed the figure legend, methods section, and main text to indicate the appropriate inoculum used for cyst counting experiments.*

Figure S1 The IVN looks normal in the PPM3C KO as stated but the image for TgPPM3C-HA does not seem to have clear tubules. Is this just the image or were there any differences between this and the parental line?

*We find variability in IVN contents or how prominent the IVN is within vacuoles formed by Pru strain parasites compared to RH strain vacuoles. Despite this, we note no gross differences in IVN formation comparing EM sections from the TgPPM3C-HA strain to those from the parental PruQ strain.*

Line 173 Consider rewording ‘processed through LC-MS/MS to obtain peptide spectra’ to ‘analysed by LC-MS/MS’ or something similar.

*We changed the text to “analyzed by LC-MS/MS” as suggested.*

Line 183 ‘more than half (79) of all differentially abundant phosphopeptides belong to proteins previously known to be secreted into the lumen of the parasitophorous vacuole (Dataset S2).’
Can this information be added to the table?

*We included a separate column in the first sheet of the S2 dataset to indicate whether a protein is known to be secreted into the PV lumen, and also changed the number of phosphopeptides stated (73) to include only those belonging to proteins known or previously reported to be secreted into the PV lumen.*

Line 206:’ In line with our initial hypothesis’ It would be helpful to remind the reader what this is.

*We re-stated the initial hypothesis as suggested.*

Fig 3D/methods – it would be helpful if you added a comment on what hypergeometric testing is -this phrase is used several times but with no description even in the methods.

*We expanded the description on how hypergeometric testing was performed in the Materials and Methods section.*

Line 212: This logic seems a bit flawed as intravacuolar components also show virulence defects in vivo so there doesn’t seem to be a reason why PPM3C targets leaving the vacuole would be more important than intravacuolar.

*We agree that this train-of-thought is not so compelling given the virulence defects observed in other parasite strains lacking intravacuolar components. We therefore removed this logic and solely describe that the Co-IP and phosphoproteomic data led us toward investigating whether defects in effector export may be evident in the deltaPPM3C strain.*

Line 237: I find this reasoning a little narrow – PPM3C modification of MYR1 could still have distinct impacts on different subsets of effectors. We understand so little about this mechanism, perhaps MYR1 phosphorylation affects its interactions with other translocation components, chaperones or effectors.

*We agree that the original reasoning, at least in this section of the manuscript, didn’t sufficiently acknowledge the possibility that altered phosphorylation status of GRA16/GRA28* ***and/or*** *MYR1 could be contributing to the export defect. We changed the wording to acknowledge this possibility.*

Line 255: th to the

*Changed*

Fig 4A/B: Please add that this is 24h infection to the legend.

*Timepoint of infection added to the legend for each figure*

Fig 4A: It is stated that you only analyse cells with one PV but the image appears to have two?

*We agree with the reviewer that based on our analysis paradigm a representative image that more clearly demonstrated a single PV should have been provided for this figure. We provided a new representative image that more clearly illustrates infection with a single PV*

Fig 4B: Is the myc staining panel only myc signal currently? I haven’t seen the signal from the parasites before.

*We included a comment in the figure legend that we interpret the signals detected within the parasite and parasite PVs to be non-specific for both the anti-Myc and anti-EZH2 antibodies used in this experiment. We have seen this before with both of these antibodies in our laboratory.*

Figure 4C: Please add uninfected and PRuQ labels to the images (as the rest are labelled).

*Labels for the images in Fig 4C and 4D were added to indicate infection with PruQ or TgPPM3C-HA, while asterisks indicate an uninfected cell. This information is also provided in the figure legend.*

Line 484: Please add magnification used for these fields of view.

*The objective lens (40X magnification) used to collect images with the slide scanner instrument was added to the Materials and Methods section, and the size of the scale bars for each representative image collected from the slide scanner was added to the appropriate figure legends.*

Fig 6: This is perhaps more suited as a supplementary figure and I think needs less attention in the discussion.

*We changed Figure 6 to Fig S5 and condensed the paragraph describing the TgPPM3C crystal structure, as suggested.*

Dataset S1
Please check column names eg ‘PruQ (ctrl) Tac’ only doesn’t describe what the number actually refers to.
Also, I found the format of empty columns with a title referring to following columns initially unclear (looks like missing data). I would suggest changing these to the row above (personal opinion only).
Combined analysis tab – Rep 1 p values showing as div/0 error
Is the ‘Reference cells from Rep2 sheet for indexing’ meant to be there?
Combined analysis results tab – p values are >10. I assume this is -log p, please label as such.

*We extensively modified the appearance of Dataset 1 and 2, incorporating all of the above suggestions (and those below), as well as clarifying any confusing columns/sheets. We appreciate the reviewer’s careful review of these datasets and apologize for these formatting issues in these datasets.*

Methods: Most of this is written short format so that it could not be easily repeated.

*The Material and Methods section has been expanded after incorporating reviewer suggestions and clarifying other details.*

Dataset S2
Remove *Absent in all control samples (candidate for mutagenesis?) from row 663
There is no legend – a description of the tabs would be helpful as its quite unclear, especially which to focus on of the normalised, cumul_normalised volcano and the final corrected tab.

*We have included more information in the description/legends for Datasets 1 and 2 to help guide the reader. We removed the “candidate for mutagenesis” comment as well.*

Data availability:
Acquired RAW files should be deposited in a dedicated mass spec open access repository, like ProteomeXchange via PRIDE, for example.

*We have contacted ToxoDB and are in the process of working with them on depositing the phosphoproteomic data from LC-MS/MS. We have deposited the data from both the S1 and S2 datasets onto the mass spectrometry open access repository Chorus under Project ID 1695. The deposition information has been added to the manuscript.*

Co-IP experiment:
1) The authors claim they did this experiment in 2 independent replicates (N=2). Could they explain how they managed to obtain individual p-values for replicate 1 and 2 (S1 Dataset) where they have no technical replicates just the co-IP and control?

*For each replicate, peptide intensity values (i.e. area under the curve for a given peptide spectrum) were log2 transformed, normalized, and imputed, as demonstrated in the S1 dataset file (see ‘Replicate 1 Analysis’ and ‘Replicate 2’ analysis sheets in the S1 dataset; equations are provided in each cell containing data within these sheets). The fold change for a particular peptide was obtained by subtracting the log transformed peptide intensity value in the TgPPM3C-HA sample by the log transformed value from the control untagged PruQ sample for each replicate (which was an imputed value if absent in the control sample). The average fold enrichment for a given protein was then estimated by averaging each peptide fold change belonging to a particular protein.*

*P-values were then obtained for each protein using a custom excel formula in which the t-distribution was calculated using the average protein fold enrichment and the log transformed peptide values to calculate the standard deviation, sample size, and degrees of freedom. p-values could only be obtained for proteins in which at least two peptides were detected.*

*We included a more thorough description of this analyses in the Materials and Methods section for clarity.*

2) How was the reported fold change cut off estimated in this experiment?
Note: in the manuscript text it is reported as FC > 1.5 whereas in the S1 Dataset it says FC > 1.

*The fold-change cutoff was arbitrarily selected (added to the manuscript text in Materials and Methods section). We apologize for the discrepancy between the manuscript text and S1 dataset – both should have indicated a FC cut-off of +1.5 for the Co-IP experiment*

3) No search engine generated identification quality parameters, such as identification scores, number of unique peptides per protein or PEPs are given in the S1 dataset.

*This information has now been provided under the “Raw Data” tabs in the S1 dataset.*

Phosphoproteome experiment:

1) How was the fold change (-/+ 1.2) cut off selected in this experiment?
Note: in the manuscript text it is reported as FC -/+ 1.2 whereas in the S2 Dataset it says FC -/+ 1.5 (Normalized results tab)

*The fold-change cutoff was arbitrarily selected (added to the manuscript text) based on previously used cutoffs for similar phosphoproteomic datasets. We again apologize for the discrepancy between the manuscript text and S2 dataset – both have been changed to a FC cut-off of -/+ 1.5*

2) The number of detected phosphopeptides is actually 5,881 (total) and 2,387 (Toxo) as per S2 Dataset/tab Phospho.

*There was an error in the original version of this manuscript, as in actuality there were 5,881 total* ***peptides*** *that were enriched by TiO_2_ (and not phosphopeptides). This has been corrected in the main text (3,896 total phosphopeptides, 1,753 parasite phosphopeptides).*

3) S2 Dataset needs some clarification:

a) No search engine generated identification and phosphosite localization parameters are reported. One can only assume that the value in parenthesis by the site info could be localization probability?

*This information has been added under the “Raw Data” tabs in the S2 dataset. The value in parenthesis indeed indicates localization probability for a given phosphopeptide, and this is now explained in the dataset file within the appropriate column headers.*

b) In several instances within different tabs (Normalized results and Norm Volcano tabs) the authors seem to use normalized phosphosite intensities (per sample) and then report the FC for a phosphopeptide. This does not seem to be correct taking into account the manuscript text, Fig 3A and S2 Dataset where authors report they performed quants and normalization on the phosphopeptide level. Please clarify.

*Phosphopeptide intensities were first log transformed, normalized by the average value of each sample, and then imputed to obtain transformed peptide intensities. After this data transformation, a separate normalization by protein abundance was then performed on the transformed data i.e. transformed phosphopeptide data was normalized by cumulative protein intensities detected in TiO_2_ flow through fractions, before assessing statistical changes. We clarified the description of this analysis in the Materials and Methods section for the reader.*

c) It would be easier for a reader to find their way around the table if the host and parasite entries were separated into different tabs. The authors could also provide the list of significantly changing Toxoplasma phosphopeptides/phosphoproteins in a separate tab since this is the most important piece of information the reader looks for when browsing that table.

*The layout of the S2 dataset has been significantly altered, as we agree and apologize for how confusing the original dataset was. The first tab that opens for the S2 dataset displays Toxoplasma phosphopeptides only, ranked by significance. The other tabs demonstrate how the data was analyzed for transparency, while the raw data sheets contain search engine identification scores for phosphopeptides enriched by TiO_2_ and proteins identified in the TiO_2_ flow through fractions.*

d) What is the blue colour coding in Normalized results and Volcano tabs?

*Blue color coding has been removed as it had no meaning.*

Reviewer #2: 1) It was difficult to understand the S1 dataset. In the combined analysis datasheet why does each phosphopeptide have the same P-value and fold-change? When a phosphopeptide was not detected in one strain but it was in the other it seems a value was imputed that was 2 SD below the mean. Is the rationale for this because this is the limit of detection? Why not use the value of the lowest detectable peptide?

*We apologize for how disorienting both the original S1 and S2 datasets were and have improved the presentation of these files. In the revised versions, we condensed the amount of sheets/tabs for both excel datasets and expanded the figure legends for both of the datasets to help guide the reader.*

*The “same p-value and fold change” described by the reviewer was an error that we missed in the old S1 or S2 datasheets from the use of reference cells for built-in equations in Microsoft Excel.*

*Regarding imputation, we used the “2 SD below the mean” approach for both the Co-IP and phosphoproteomic datasets to transform the data toward generating as close to a normal distribution as possible, so that t-tests could be performed under this assumption. Using the lowest detectable peptide might be another reasonable approach for imputation, but we believe the data would less likely represent a normal distribution using this method, and thus results from t-tests less valid.*

Reviewer #2: 2) Fig. 4a states that the data were collected from 3 technical replicates. Therefore, it appears this experiment was only performed once. The authors should repeat the experiment and present averages from at least 3 independent experiments.

*We repeated the experiments and in the revised manuscript present the data from three independent experiments as three violin plots for each figure, to demonstrate the spread of the data.*

Reviewer #3: Fig 3C,D- proteins with increase phosphopeptides in the TgPPM3C KO do not need to be direct substrates of TgPPM3C. Thus, not finding a consensus sequence within them, does not rule out that TgPPM3C has a consensus sequence.

*We thank the reviewer for pointing out this possibility. We added sentences regarding this point both in the Results and Discussion sections.*
