# Supplementary material for: Toxoplasma gondii PPM3C, a secreted protein phosphatase, affects parasitophorous vacuole effector export
Source: PLoS Pathog. 2020 Dec 28;16(12):e1008771. doi: 10.1371/journal.ppat.1008771 (PMC7793252; doi:10.1371/journal.ppat.1008771)
Supplement: Attachment — Submitted filename: Response Letter final version V3.docx [file ppat.1008771.s010.docx]

**Reply to the Review**

*We first would like to thank the editor and reviewers for their thoughtful comments, and for considering this work as valuable and of interest to the Toxoplasma field. We were pleased that the reviewers believed that we had addressed their previous comments. We have revised the manuscript to address the comments of the editor and added additional data on uninfected cells to the figures as suggested. We have provided a detailed response to the various reviewer's comments below in 12pt italicized font. We believe that the new experimental data and revisions have significantly improved this manuscript and that this work merits publication in PLoS Pathogens. All of the authors agree with submission of this revision and in the event of publication the authors assign copyright in accordance with the instructions to authors.*

**Response to the Editor's comments**:

As you will read, the reviewers were in general agreement that you addressed most of their concerns. Reviewer #3 was, however, still concerned about the serine to proline mutants. While I share some of these concerns, I feel that since the mutations lies in a disorganized domain of the protein it is difficult to predict the impact of these substitutions. But addressing this would require a substantial level of structure/function assays that, due to COVID, I am afraid would take an overly significant amount of time to complete. I do, however, agree with the reviewer's suggestion about showing images of and quantifying uninfected nuclei.

*We appreciate the editor's comment that we have addressed the previous concerns. We agree with the editor that it is difficult to predict the impact of the aa substitutions in the disorganized domain of this protein. We appreciate the editor recognizing the substantial amount of additional work the suggestion of repeating the experiments with the additional mutants suggested by reviewer 3. In addition, we believe the aa substitutions we have performed are the correct modifications to address the experimental issues discussed in this paper. As suggested by reviewer 3 and the editor we have included images of uninfected nuclei and also provided data (Figure 4) quantifying the fluorescence seen in uninfected nuclei.*

**Response to the reviewer’s comments:**

**Part I Summary (**Please use this section to discuss strengths/weaknesses of study, novelty/significance, general execution and scholarship)

Reviewer #1: In the revised version the authors have addressed my concerns and I think this is a very interesting contribution to our understanding of the development of the intracellular niche of Toxoplasma in a host cell. The role of differential phosphorylation of secreted proteins remains an enigma and the role of phosphatases has previously not been addressed. Of particular interest is the specific effect of an export defect of only some, but not all proteins that are exported further into the host cell. This argues for a complex pattern of protein regulation of secreted proteins.

Reviewer #2: The authors have addressed all my comments.

*We appreciate the comments that we have addressed the previous concerns and that this is an interesting contribution to the literature.*

Reviewer #3: The authors have addressed my concerns in terms of improving the staining for TgPPM3C; addressing invasion, replication, and egress; and performing their assays with a clonal, endogenously tagged GRA16 and a transiently expressing epitope tagged TgIST. Unfortunately, they chose to not address the concerns about using a proline substitute for the phospoablative GRA16. Basically- if your positive control doesn’t act as a positive control, that is a problem. This issue calls into question the results of the phosphomimetic, which is the lynchpin to the novelty and impact of this work, especially given the Cyagen study which has significantly different findings (which may or may not relate to be strain specific differences)..

*We appreciate that we have addressed the majority of the concerns of this reviewer. However, we respectfully disagree with the notion that phosphoablative GRA16 is a “positive control”; this wording is not used in the manuscript, as the field does not yet sufficiently understand the rules governing the export of these effector proteins (e.g. Are serine-rich repeats specifically recognized by the translocon, or are tandem repeats with variable amino acid content sufficient for recognition?; Does the fully assembled translocon interact with non-phosphorylated serine with a greater affinity over other amino acids in tandem repeat regions?; etc.). The phosphoablative GRA16 construct merely provides an additional comparison to the phosphomimetic GRA16 experiment. Phosphomimetic GRA16 clearly demonstrates a significant export defect compared to both the wild type and the phosphoablative GRA16, which we believe provides excellent evidence for the proposed model implicating dephosphorylation of GRA16 as an important step in the export process.*

**Part II – Major Issues: Key Experiments Required for Acceptance (**Please use this section to detail the key new experiments or modifications of existing experiments that should be absolutely required to validate study conclusions. Generally, there should be no more than 3 such required experiments or major modifications for a "Major Revision" recommendation. If more than 3 experiments are necessary to validate the study conclusions, then you are encouraged to recommend "Reject".)

Reviewer #1: No major issues.

Reviewer #2: None.

*We appreciate that all of the previous issues were addressed in our revision.*

Reviewer #3: You will need to re-do the phosphoablative studies with a GRA16 uses alanine or glutamine substitutions for serine.

*We feel that sufficient rationale is provided in the text on the use proline instead of other amino acids substitutions to mimic a phosphoablative mutation. The goal was to maintain protein intrinsic disorder in GRA16, which is known to be important for the proper export of this and other secreted effector proteins. The use of either alanine or glutamine would probably alter the intrinsic disorder as noted in the rationale in the paper. We appreciate the editor recognizing the substantial amount of additional work the suggestion of repeating the experiments with the additional mutants. We agree with the editor that it is difficult to predict the impact of these aa substitutions in the disorganized domain of this protein.*

**Part III – Minor Issues: Editorial and Data Presentation Modifications** (Please use this section for editorial suggestions as well as relatively minor modifications of existing data that would enhance clarity)

Reviewer #1: I am happy with the current version. It may be worthwhile to discuss these results in the light of a recent publication (Young et al., 2020 mSphere) that describes differential phosphorylation between the Tachyzoite and Bradyzoite stage- and if proteins that are targets of PPM3C are found among these, maybe implying a wider role for this phosphatase in latent stage parasites.

The one experiment I would love to see - although not a request for publication of this manuscript as slightly beyond the scope - whether PPM3C KOs have a defect in cystwall formation in vitro. This could illuminate whether PPM3C has an additional defect in cyst formation.

*We thank the reviewer for these suggestions. We have incorporated an additional discussion on Young et al., 2020 mSphere as it relates to PPM3C findings (see highlighted text in the discussion section). We also updated the phosphoproteomic S2 dataset to include the new short name annotation for hypothetical protein TGME49_289540, referred to as strand forming protein 1 (SFP1) in Young et al. Notably, both SFP1 and GRA29 (also characterized in Young et al., 2020) were detected as more abundant in the deltaPPM3C strain, suggesting these proteins are also phosphoprotein substrates of this particular phosphatase.*

*Regarding defects in cyst wall formation, we will keep this line of inquiry in mind as we consider the next steps in understanding PPM3C function in both life stages. We did assess spontaneous bradyzoite differentiation under tachyzoite growth conditions in the deltaPPM3C strain, but did not note any significant differences compared to the parental strain.*

Reviewer #2: The differences in Fig. 4B and 5B don't seem very large and the volcano plots largely overlap between the different groups. It seems surprising that these are so highly significant. Maybe the authors can double check their analyses.

*We double-checked the analysis as suggested, and also performed a more stringent parametric test (One-way ANOVA) to compare groups in both of these experiments, which might be considered appropriate with the sample sizes obtained for each independent experiment (n > 30). This analysis still yields significant differences when comparing the means from independent experiments across each group. While it may be challenging to appreciate differences in means among the violin plots across each group, we favor violin plot presentation to demonstrate the spread of all observations in each experiment.*

Reviewer #3: Fig 2 B: please quantify your plaque assays as well (i.e. not just plaque size but also number)

.

*We have included the number of plaques recorded from multiple wells above each violin plot for each independent experiment. The figure legend for 2B has also been modified to indicate this.*

Fig 4A: Gra24- which they are using as a negative control, for both PruQ and delta TgPPM3C, has an intensity at the level of uninfected cells (see Fig 4B) or cells in which the GRA protein is not secreted (intensity level for Gra28 in TgPPM3C parasites). This suggests they are not detecting GRA24 secretion in PruQ or TgPPM3C, which is consistent with the provided IFA. To address this issue, the authors should provide an image with nuclei from infected and uninfected host cells (akin to 4B COMP image, 4C, and 4D).

*The uninfected intensity values reported for the c-Myc IFA experiments are challenging to compare to the intensity values recorded for the exported effectors as longer exposure times were used to detect c-Myc fluorescent intensity signals, resulting in greater amounts of background fluorescent intensity (i.e. larger intensity values for uninfected nuclei). We included an image of uninfected cells for the GRA24 IFA, which clearly shows the absence of GRA24 signal compared to the infected fibroblast. In addition, we have provided violin plots of the intensity of signal seen in uninfected cells in these experiments. We believe this clearly demonstrates that GRA24 export is indeed being detected in these experiments. Please also note that GRA28 secretion is still observed in deltaPPM3C parasites.*

Line 290 – 297: The results with c-Myc are more consistent with ref 25 rather than ref 21

*Lines 292-297 describe that deltaPPM3C parasites can still induce host c-Myc, as observed and published in Ref 21 (Cygan et al., mSphere, 2020). For this reason, we cite Ref 21 in this part of the manuscript.*

Line 312: For improved accuracy, the phrase “GRA16 expressed ectopically” should be revised to “GRA16 transiently ectopically expressed” (the images in 4A were generated by transient expression as far as I can tell

*We rephrased this wording as suggested.*

Line 317 – 330/Fig S3B: How many phosphate groups are being added to GRA16? As each phosphate group adds ~80 Da, so the bands in S3B don’t seem quite right. (The lower band in the gel from the extracellular parasites seems reasonable, but the upper band seems extreme)

*We detected five different GRA16 phosphorylation sites in deltaPPM3C parasites. The mass of these phosphate groups alone would indeed not account for the size discrepancy observed for the >55kDa band. However, phosphorylation is known to induce “gel-shifts”on SDS-PAGE, which retard the migration of the protein to a greater magnitude than what would be expected solely from the molecular weight of this post translational modification (see Wegener and Jones, J Biol Chem, 1984 for an example with the protein phospholamban). Any of the GRA16 phosphomodifications might impact the tertiary structure of GRA16 and/or conceivably affect SDS binding to the protein, thus impacting migration. We believe the influence of alkaline phosphatase treatment on the larger band in both the parental and deltaPPM3C strains provides good evidence that this band may well represent “gel-shifted” phospho-GRA16.*

Line 356 – 369/Fig 5C: I’m not sure how these experiments show that the mutations in amino acid content in GRA16 don’t affect protein stability.

*Probably the strongest evidence for the lack of protein stability differences in mutagenized and wild type GRA16 protein would be direct measurements of protein half-life. However, as our laboratory does not have expertise in this area, we opted to make GRA16 export measurements in the deltaPPM3C background. These measurements demonstrated no differences in host nuclear GRA16 intensity, implying that similar amounts of GRA16, mutagenized or nonmutagenized, are available to be recognized and exported by the effector translocon in the absence of PPM3C. While it is conceivable that the absence of PPM3C may have affected “GRA16 stabilizing factor(s)” within the parasitophorous vacuole in these experiments, we believe this is less likely.*
